# Supplementary figures and images for: Neotropical bats as sentinels for emerging zoonoses in Central America: A case study identifying Trypanosoma cruzi in bats from Belize using metagenomic next-generation sequencing
Source: PLoS Negl Trop Dis. 2026 Jul 23;20(7):e0013851. doi: 10.1371/journal.pntd.0013851 (PMC13395375; doi:10.1371/journal.pntd.0013851)

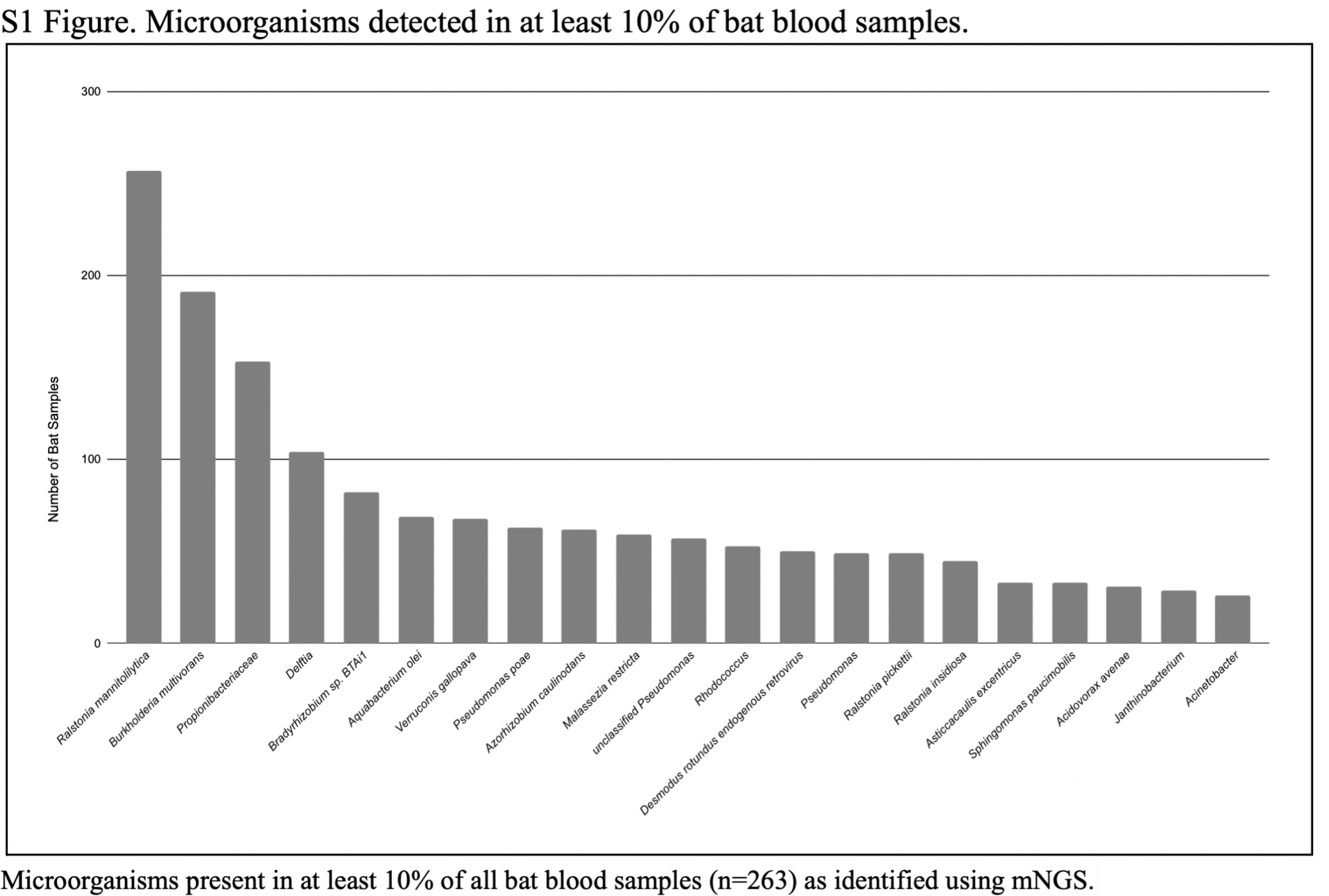

Supplement: S1 Fig — Microorganisms present in at least 10% of all bat blood samples (n = 263) as identified using mNGS. (TIF) [file pntd.0013851.s001.tif]
