# Supplementary material for: Neotropical bats as sentinels for emerging zoonoses in Central America: A case study identifying Trypanosoma cruzi in bats from Belize using metagenomic next-generation sequencing
Source: PLoS Negl Trop Dis. 2026 Jul 23;20(7):e0013851. doi: 10.1371/journal.pntd.0013851 (PMC13395375; doi:10.1371/journal.pntd.0013851)
Supplement: S1 Table — (DOC) [file pntd.0013851.s002.doc]

S1 Table. Bat species collected in Belize, Central America from which samples were tested using metagenomic next-generation sequencing.

| **Species** | **Number of Specimens** |
| --- | --- |
| *Artibeus intermedius* | 6 |
| *Artibeus jamaicensis* | 38 |
| *Carollia perspicillata* | 6 |
| *Carollia sowelli* | 23 |
| *Dermanura phaeotis* | 6 |
| *Desmodus rotundus* | 43 |
| *Eptesicus furinalis* | 5 |
| *Gardnerycteris keenani* | 2 |
| *Glossophaga mutica* | 14 |
| *Glossophaga soricina* | 5 |
| *Lasiurus ega* | 5 |
| *Molossus alvarezi* | 4 |
| *Molossus nigricans* | 14 |
| *Myotis elegans* | 3 |
| *Pteronotus fulvus* | 10 |
| *Pteronotus mesoamericanus* | 25 |
| *Rhynchonycteris naso* | 6 |
| *Saccopteryx bilineata* | 9 |
| *Sturnira parvidens* | 22 |
| *Uroderma convexum* | 17 |
| **Total** | **263** |
