# Supplementary material for: Neotropical bats as sentinels for emerging zoonoses in Central America: A case study identifying Trypanosoma cruzi in bats from Belize using metagenomic next-generation sequencing
Source: PLoS Negl Trop Dis. 2026 Jul 23;20(7):e0013851. doi: 10.1371/journal.pntd.0013851 (PMC13395375; doi:10.1371/journal.pntd.0013851)
Supplement: S3 Table — C- water, no template control; 1:100 C + , positive control; 1:1,000 C + , positive control; OBZB-00171, negative control; OBZB-00095; OBZB-00121; OBZB-00154; OBZB-00161; OBZB-00165; OBZB-00254. (DOC) [file pntd.0013851.s004.doc]

S3 Table. Quantitative PCR amplification plot results for controls and samples that tested positive for *Trypanosoma cruzi* using metagenomic next-generation sequencing.

| **Sample ID** | **DNA (ng/µL)** | **RNA (ng/µL)** | **Ct** | **Result** |
| --- | --- | --- | --- | --- |
| C- water | N/A | N/A | Undetermined | Negative |
| 1:100 C+ | 0.246 | Below detectable range | 15.96 | Positive |
| 1:1000 C+ | Below detectable range | Below detectable range | 18.68 | Positive |
| OBZB-00171 (Trypanosoma C-) | 0.0353 | 0 | Undetermined | Negative |
| OBZB-00095 | 0.113 | 1.71 | 41.03 | Positive |
| OBZB-00121 | 0.139 | Below detectable range | Undetermined | Negative |
| OBZB-00154 | 0.0853 | Below detectable range | 33.86 | Positive |
| OBZB-00161 | 0.339 | 2.16 | 38.96 | Positive |
| OBZB-00165 | 0.0787 | Below detectable range | Undetermined | Negative |
| OBZB-00254 | 0.085 | Below detectable range | 37.90 | Positive |

C- water, no template control, 5.5 μL H_2_O; 1:100 C+, positive control, 2 μL 1:100 dilution *Trypanosoma cruzi* gDNA; 1:1,000 C+, positive control, 2 μL 1:1,000 dilution *Trypanosoma cruzi* gDNA; OBZB-00171, negative control, 2.0 μL DNA from sample positive for *Trypanosoma* genus via mNGS; OBZB-00095, 2.0 μL DNA from sample positive for *Trypanosoma cruzi* via mNGS; OBZB-00121, 2.0 μL DNA from sample positive for *Trypanosoma cruzi* via mNGS; OBZB-00154, 2.0 μL DNA from sample positive for *Trypanosoma cruzi* via mNGS; OBZB-00161, 2.0 μL DNA from sample positive for *Trypanosoma cruzi* via mNGS; OBZB-00165, 2.0 μL DNA from sample positive for *Trypanosoma cruzi* via mNGS; OBZB-00254, 2.0 μL DNA from sample positive for *Trypanosoma cruzi* via mNGS.
