# Supplementary material for: Neotropical bats as sentinels for emerging zoonoses in Central America: A case study identifying Trypanosoma cruzi in bats from Belize using metagenomic next-generation sequencing
Source: PLoS Negl Trop Dis. 2026 Jul 23;20(7):e0013851. doi: 10.1371/journal.pntd.0013851 (PMC13395375; doi:10.1371/journal.pntd.0013851)
Supplement: S4 Table — Databases totaling 726 GB retrieved from https://github.com/ncbi/blast_plus_docs#blast-databases. (DOC) [file pntd.0013851.s005.doc]

S4 Table. NCBI databases BLAST+ used to confirm microorganism identities. Databases totaling 726 GB retrieved from <https://github.com/ncbi/blast_plus_docs#blast-databases>.

| **Databases** |
| --- |
| -rw-r--r-- 1 9011 xymon  1.2M Jul  5  2022 nt.68.nnd  -rw-r--r-- 1 9011 xymon  1.8M Jul  5  2022 nt.68.nin  -rw-r--r-- 1 9011 xymon   26M Jul  5  2022 nt.68.nhr  -rw-r--r-- 1 9011 xymon   501 Jul  5  2022 nt.nal  -rw-r--r-- 1 9011 xymon   57K Jul  5  2022 nt.68.nhi  -rw-r--r-- 1 9011 xymon  2.5M Jul  5  2022 nt.68.nhd  -rw-r--r-- 1 9011 xymon  1.8G Jul  5  2022 nt.nos  -rw-r--r-- 1 9011 xymon  4.4G Jul  5  2022 nt.ndb  -rw-r--r-- 1 9011 xymon  965M Jul  5  2022 nt.not  -rw-r--r-- 1 9011 xymon  332M Jul  5  2022 nt.nto  -rw-r--r-- 1 9011 xymon  175M Jul  5  2022 nt.ntf  -rw-rw-r-- 1 9011 xymon   16M Jul  8  2022 taxdb.bti  -rw-rw-r-- 1 9011 xymon  152M Jul  8  2022 taxdb.btd  -rw-r--r-- 1 root root     47 Jul  9  2022 nr.00.tar.gz.md5  -rw-r--r-- 1 root root     47 Jul  9  2022 nr.01.tar.gz.md5  -rw-r--r-- 1 root root     47 Jul  9  2022 nr.02.tar.gz.md5  -rw-r--r-- 1 root root     47 Jul  9  2022 nr.03.tar.gz.md5  -rw-r--r-- 1 root root     47 Jul  9  2022 nr.04.tar.gz.md5  -rw-rw-r-- 1 9011 xymon   748 May 26  2022 16S_ribosomal_RNA.nni  -rw-rw-r-- 1 9011 xymon  176K May 26  2022 16S_ribosomal_RNA.nnd  -rw-rw-r-- 1 9011 xymon  1.2M May 26  2022 16S_ribosomal_RNA.ndb  -rw-r--r-- 1 root root    38M Jun 14  2022 16S_ribosomal_RNA.tar.gz  -rw-r--r-- 1 root root     59 Jun 14  2022 16S_ribosomal_RNA.tar.gz.md5  -rw-r--r-- 1 root root    33M Jun 14  2022 18S_fungal_sequences.tar.gz  -rw-r--r-- 1 root root     62 Jun 14  2022 18S_fungal_sequences.tar.gz.md5  -rw-r--r-- 1 root root    33M Jun 14  2022 28S_fungal_sequences.tar.gz  -rw-r--r-- 1 root root     62 Jun 14  2022 28S_fungal_sequences.tar.gz.md5  -rw-r--r-- 1 root root   658M Jun 14  2022 Betacoronavirus.00.tar.gz  -rw-r--r-- 1 root root     60 Jun 14  2022 Betacoronavirus.00.tar.gz.md5  -rw-r--r-- 1 root root   414M Jun 14  2022 Betacoronavirus.01.tar.gz  -rw-r--r-- 1 root root     60 Jun 14  2022 Betacoronavirus.01.tar.gz.md5  -rw-r--r-- 1 root root   422M Jun 14  2022 Betacoronavirus.02.tar.gz  -rw-r--r-- 1 root root     60 Jun 14  2022 Betacoronavirus.02.tar.gz.md5  -rw-r--r-- 1 root root   361M Jun 14  2022 Betacoronavirus.03.tar.gz  -rw-r--r-- 1 root root     60 Jun 14  2022 Betacoronavirus.03.tar.gz.md5  -rw-r--r-- 1 root root   469M Jun 14  2022 Betacoronavirus.04.tar.gz  -rw-r--r-- 1 root root     60 Jun 14  2022 Betacoronavirus.04.tar.gz.md5  -rw-r--r-- 1 root root   495M Jun 14  2022 Betacoronavirus.05.tar.gz  -rw-r--r-- 1 root root     60 Jun 14  2022 Betacoronavirus.05.tar.gz.md5  -rw-r--r-- 1 root root   422M Jun 14  2022 Betacoronavirus.06.tar.gz  -rw-r--r-- 1 root root     60 Jun 14  2022 Betacoronavirus.06.tar.gz.md5  -rw-r--r-- 1 root root    34M Jun 14  2022 ITS_RefSeq_Fungi.tar.gz  -rw-r--r-- 1 root root     58 Jun 14  2022 ITS_RefSeq_Fungi.tar.gz.md5 |
